# Comparison of the efficacy of parent-mediated NDBIs on developmental skills in children with ASD and fidelity in parents: a systematic review and network meta-analysis

**DOI:** 10.1186/s12887-024-04752-9

**Published:** 2024-04-25

**Authors:** Yuling Ouyang, Junyan Feng, Tiantian Wang, Yang Xue, Zakaria Ahmed Mohamed, Feiyong Jia

**Affiliations:** 1 Department of Developmental and Behavioral Pediatrics, the First Hospital of Jilin University, Changchun, 130021 China; 2 School of Nursing, Jilin University, Changchun, 130021 China

**Keywords:** Autism spectrum disorder, Parent fidelity, NDBIs, Parent-mediated intervention

## Abstract

**Background:**

Recently, studies on behavioral interventions for autism have gained popularity. Naturalistic Developmental Behavior Interventions (NDBIs) are among the most effective, evidence-based, and widely used behavior interventions for autism. However, no research has been conducted on which of the several NDBI methods is most effective for parents and children with autism spectrum disorders. Therefore, we conducted a network meta-analysis to compare the specific effects of each type of parental-mediated NDBI on children’s developmental skills and parent fidelity.

**Methods:**

PubMed, Embase, Cochrane Library, Medline, Web of Science, China National Knowledge Infrastructure (CNKI), CINAHL, and Wanfang databases were searched from inception to August 30, 2023. A total of 32 randomized controlled trial studies that examined the efficacy of different NDBIs were included.

**Results:**

Parents of children with ASD who received Pivotal Response Treatment (PRT) reported significant improvements in their children’s social skills (SUCRA, 74.1%), language skills (SUCRA, 88.3%), and parenting fidelity (SUCRA, 99.5%). Moreover, parents who received Early Start Denver Model (ESDM) reported significant improvements in their children’s language (SMD = 0.41, 95% CI: 0.04, 0.79) and motor skills (SMD = 0.44, 95% CI: 0.09, 0.79). In terms of the efficacy of improving parent fidelity, the results showed that the Improving Parents as Communication Teachers (ImPACT) intervention significantly improved parent fidelity when compared with the treatment-as-usual group (TAU) (SMD = 0.90, 95% CI: 0.39, 1.42) and the parental education intervention (PEI) (SMD = 1.10, 95% CI:0.28, 1.91).There was a difference in parent fidelity among parents who received PRT(SMD = 3.53, 95% CI: 2.26, 4.79) or ESDM(SMD = 1.42, 95% CI: 0.76, 2.09) training compared with PEI.

**Conclusion:**

In conclusion, this study revealed that parents can achieve high fidelity with the ImPACT intervention, and it can serve as an early first step for children newly diagnosed with ASD. It also showed that parent-mediated ESDM is effective in improving language and motor skills for children with ASD and can be used as part of the second stage of parent training. Parent-mediated PRT can also be used as a third stage of parent training with sufficient training intensity to further improve language, social, and motor skills.

**Supplementary Information:**

The online version contains supplementary material available at 10.1186/s12887-024-04752-9.

## Background

Autism spectrum disorder (ASD) is a neurodevelopmental disorder characterized by social communication impairments and restricted, repetitive behaviors [1]. Early intervention is often strongly recommended for young children with autism to facilitate developmental skills in key areas to promote positive long-term outcomes [2]. There are many types of early childhood interventions recommended for this population and NDBIs(naturalistic developmental behavioral intervention)are among the most effective, evidence-based, and widely used early childhood interventions for autism [3].

NDBIs are behavioral interventions that combine developmental psychology principles with those of applied behavior analysis (ABA). This method involves sharing control between the child and the therapist, utilizing natural contingencies, and utilizing various behavioral strategies to teach skills that are developmentally appropriate and prerequisite [3]. There are several types of NDBIs [4–7] including PRT (Pivotal Response Treatment), ESDM (Early Start Denver Model), ImPACT (Improving Parents as Communication Teachers), JASPER (Joint Attention, Symbolic Play, Engagement, and Regulation), ESI (Early Social Interaction), RIT (Reciprocal Imitation Training), Social ABCs, CPMT (Cooperative Parent-Mediated Therapy), which not only follow NDBI principles, but have their own characteristics in different functional domains as well.

Grounded in Bronfenbrenner’s [8] ecological systems theory, parents play a crucial role in the early interventions provided to young children with disabilities, helping foster the child’s growth and development [9]. Empowering families by coaching parents can allow families to play a greater role in promoting children’s skill development [10]. Through the parent-mediated NDBI approach, parents have more opportunities to intervene with their children, which increases the intensity of intervention and can help children maintain skills [11]. At the same time, parents can help their children generalize skills in more new scenarios [12]. Most NDBIs include a parent intervention component. In JASPER, PRT, EMT and ImPACT, parents are the main agents of intervention, and in ESDM, family intervention is to enhance the intervention effect of the therapist [13]. Therefore, parent-mediated NDBI is a very promising intervention model.

Many studies have demonstrated that parent-mediated NDBI is effective [5, 14], however, parent-mediated NDBIs do not have significant effects in all developmental skills [15–17]. Several reasons may explain this. (1) Autistic children have different developmental characteristics. Since the developmental level, family environment, and severity of symptoms of each autistic child are different, there is also extreme heterogeneity in different developmental skills among children with ASD [18]. (2) Various NDBI have different focuses. PRT emphasizes that interventionists master intervention skills in “pivotal” areas which are designed to target motivation and maintain strong treatment fidelity; ESDM is typically used in children with ASD around the ages of 2 to 5 years old, and is a comprehensive intervention that targets developmental milestones [11, 19]; JASPER is a low intensity intervention for very young children with ASD and older prelinguistic individuals with ASD, focusing particularly on the foundations of social-communication, especially joint attention and play [20]; ImPACT is a short-term parent education program focused on teaching social communication to children with ASD or developmental language delay [21]; ESI is a comprehensive and family-centered model for toddlers with ASD and their families [22, 23]; RIT emphasizes the social role of imitation [24]; Social ABCs is an on-site parental intervention training model, the core content includes functional language and positive emotion sharing [25]; CPMT is a parent-mediated intervention method that emphasizes cooperative interaction [7]. Many studies have discussed the commonalities of NDBIs [4, 26], but no studies have examined the differences of NDBIs using quantitative method. (3) Parents receive training of varying intensity. Studies have shown that the intensity of direct intervention given to autistic children by therapists is not related to the child’s later outcomes [27, 28]. However, no studies have examined whether increasing the intensity of parent training will indirectly affect the efficacy of interventions for children.

Since parents have the opportunity to intervene in natural settings with their autistic children, family intervention needs to be recognized as an important component of early intervention. Therefore, it is imperative that clinicians determine which NDBI is most appropriate for the families of children with ASD. However, no research has been conducted on which of the several NDBI methods are most effective for parents and children with ASD, thus significantly limiting the effectiveness of the NDBI. We, therefore conducted a systematic review and network meta-analysis of randomized controlled trials (RCTs) to compare the effects of different types of parent-mediated NDBI on different developmental domains (language, social and motor skills) of children as well as parenting fidelity. We hoped this meta-analysis would help clinicians determine which NDBIs is the most appropriate for families of children with ASD.

## Methods

### Search strategy

As of August 9, 2023, a total of nine databases were searched to identify studies eligible for the Project AIM meta-analysis, including PubMed, Cochrane Library, Embase, Medline, China National Knowledge Infrastructure (CNKI), CINAHL, Web of Science and Wanfang databases. The search strategy was “autistic”, “autism”, “Asperger” and “parent”, “caregiver”, “mother” and “RCT”, “randomized clinical trial”, “randomized controlled trial”. The details of the search strategy were provided in Appendix S1.

### Selection criteria

The selection criteria were based on PICOS principle, specific criteria were given in Table 1. In our study, the control group was divided into 2 groups, the treat as usual (TAU) group, and the parent education intervention (PEI) group. The PEI group and the experimental group used the same intervention method, but the time therapist guided for parents did not exceed 50% of the experimental group [29]. NDBI methods with a total number of studies more than 2 in this meta-analysis are classified as Common NDBI, and NDBI methods with a total number of studies less than or equal to 2 are classified as Uncommon NDBI. Referring to previous similar studies, the outcome of any measure of ASD children and parents was incorporated, including the skills of language, social, motor and parent fidelity.

### Data extraction and quality assessment

Relevant data were extracted independently by two researchers by using standardized extraction forms. Across all rated items for included studies, agreement on calculations was 90%. Disagreements were attributable to (1) miscalculations, (2) unidentified outcome.

Risk of bias was rated using the Risk of Bias 2.0 (RoB 2.0), which were divided into five domains, including randomization process, deviations from the intended interventions, missing outcome data, measurement of the outcome, and selection of the reported results. Each domain could be ranked as three levels of risk levels, like “low risk”, “high risk” or “some concerns”. The evaluation was conducted independently by two researchers.

All disagreements were solved by consensus, and where consensus was not achieved, assistance was sought from the statistical consultation clinic of the First Hospital of Jilin University.

### Statistical analyses

We carried out network meta-analysis using Stata statistical software 17.0 with Stata packages network, mvmeta, metareg, metan, metafunnel and metaninf. Publication bias was examined using funnel plot analysis [30]. Effect size heterogeneity was examined using I^2^ as a measure of the proportion of true heterogeneity to total effect size variance. We used the random-effects model rather than the fixed-effects model to calculate mean difference effect size of parent training on the outcome of child and parent because random-effects models are more conservative [31]. Sensitivity analysis was used to assess the stability of the meta-analysis results on the *P* value of the forest plot and the ranking of the SUCRA plot. During sensitivity analysis we excluded studies with a sample size of less than 20 and Uncommon NDBIs whose total number of studies is less than 3.

**Table 1:** Selection criteria

Variable	Inclusion Criteria	Exclusion Criteria
Participants	Children who meet diagnostic criteria for ASD in DSM-5 or ADOS-2 and their parents	Children without a diagnosis of ASD
Intervention	Using NDBIs	Other interventions; No parents were involved in intervention training
Comparison	Treat as usual or wait for treatment, or take the same NDBI method as the experimental group but the time therapist guided for parents did not exceed 50% of the experimental group	Intervention mediated by research staff
Outcome	Any measure of developmental skills in children, including the skills of language, social, motor in children with ASD; Any measure of parent fidelity	EEG, imaging tests or blood tests
Study design	Treatment-control case	Single case; The total number of subjects is less than 10

## Results

### Study identification and selection

**Fig. 1:**
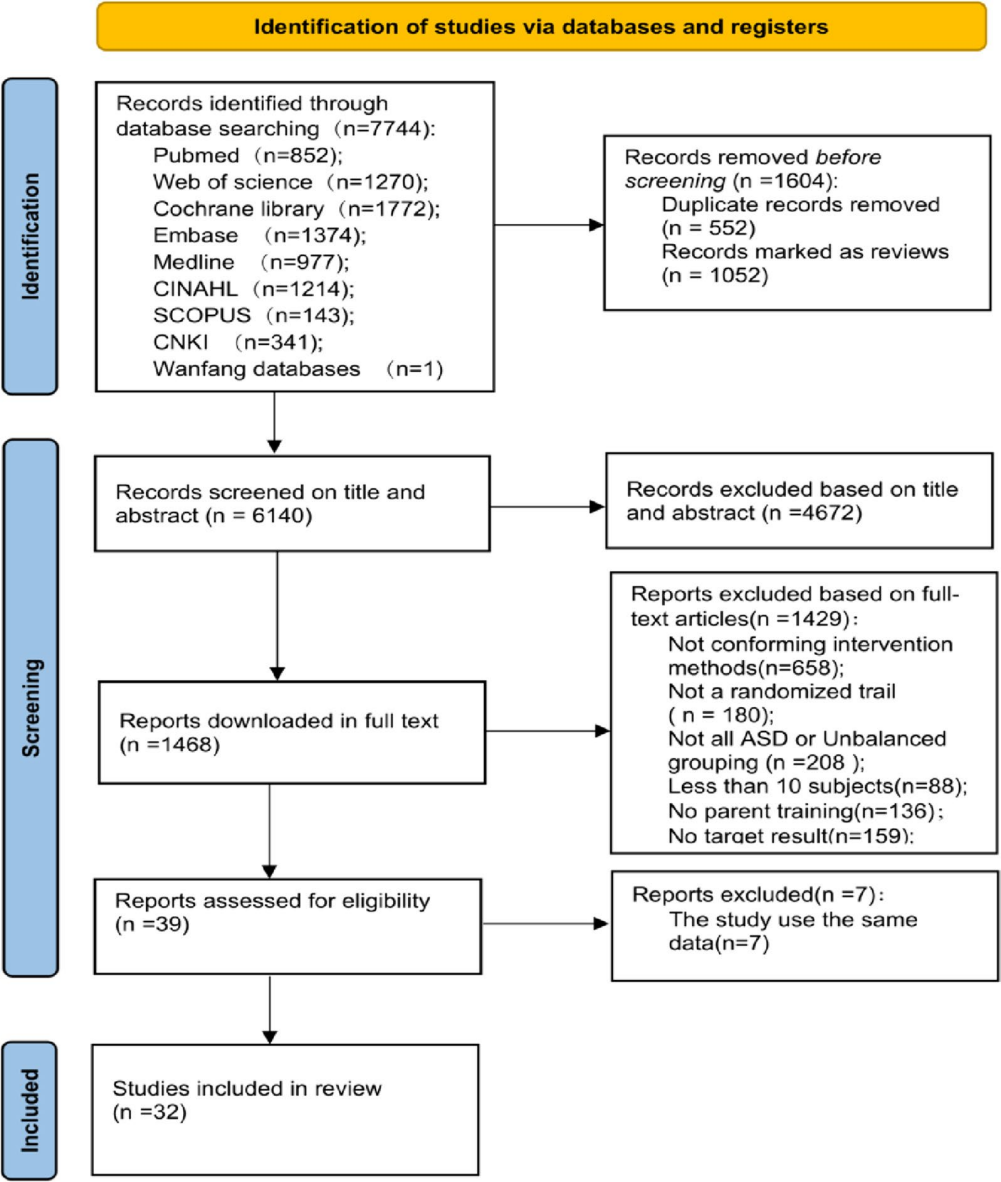
Flow diagram of the search and selection of the included studies

The initial search yielded 7744 records. No additional records were identified from other sources of the 7744 identified studies, 1604 references were duplicates. After screening the title/abstracts and full-text information, 4672 and 1429 studies were excluded, respectively. Finally, 32 studies that met the inclusion criteria were included. The study selection flow chart is shown in Fig. 1.

**Table 2:** Characteristics of the included studies

Study	Country	Sample size		Experimental group			Control group	Outcome	
		Experimental / Control		Training method	Duration	Total hours	Training method	Assessment Tools	Evaluators
PRT vs. TAU									
Gengoux et al. 2019	USA	23	20	Individual courses	24w	15 h	NP	MSEL、SRS	Researchers
Wang et al. 2023	CHN	15	15	Individual courses	8w	14 h	Individual courses	SCQ、VB-MAPP	Researchers
Vernon et al. 2019	USA	12	11	Individual courses	26w	52 h	NP	ADOS、PLS、MSEL	Researchers
PRT vs. PEI									
Drapalik et al. 2022	USA	9	6	Individual courses	10w	7 h	Self-study	BIRS	Parents
Hardan et al. 2015	USA	25	22	Group	12w	16 h	Group	VABS、PLS、SRS	Researchers
ESDM vs. TAU									
Rogers et al. 2012 [11]	USA	49	49	Individual courses	12w	12 h	NP	ADOS、MSEL、ESDM Parent Fidelity Tool	Researchers
Rogers et al. 2019	USA	55	63	Individual courses	108w	108 h	NP	ADOS、MSEL	Researchers
Zhou et al. 2018 [32]	CH N	23	20	Individual courses + Group	26w	47 h	NP	GDC-C、ADOS	Researchers
Di et al. 2020	CHN	33	33	Group	10w	35 h	NP	CARS	Parents
Chiang et al. 2023 [33]	CHN	21	24	Group	24w	12 h	NP	ADOS 、MSEL、ESDM Parent Fidelity Tool	Researchers
Dawson et al. 2010	USA	24	21	Individual courses	96w	48 h	NP	MSEL、ADOS	Researchers
ESDM vs. PEI									
Qu et al. 2022 [34]	CHN	18	14	Group	12w	18 h	Self-study	Program Evaluation Survey	Researchers
Rogers et al. 2018	USA	21	24	Individual courses	12w	36 h	Individual courses	P-ESDM fidelity、MSEL、ADOS	Researchers
Vismara et al. 2018	USA	14	10	Individual courses	12w	18 h	Individual courses	P-ESDM fidelity	Researchers
ImPACT vs. TAU									
Yoder et al. 2021 [21]	USA	49	48	Individual courses	12w	7.2 h	NP	MSEL、ADOS、Project ImPACT fidelity checklist	Researchers
Li et al. 2022	CHN	35	33	Individual courses + Group	8w	12 h	NP	SRS	Parents
Akhani et al. 2021	IRL	19	21	Individual courses + Group	12w	18 h	NP	CARS、FEAS	Researchers
Stahmer et al. 2020	USA	11	12	Individual courses	12w	30 h	Individual courses	PIT Fidelity、VABS	Researchers
ImPACT vs. PEI									
Ingersoll et al. 2016 [35]	USA	14	13	Individual courses	12w	12 h	Self-study	Project ImPACT fidelity checklist、VABS	Researchers
JASPER vs. PEI									
Kasari et al. 2014 [36]	USA	60	52	Individual courses	12w	24 h	Group	The Early Social Communication Scales、Caregiver’s quality of fidelity	Researchers
Carr et al. 2015	USA	63	54	Individual courses	12w	24 h	Group	Parent Adherence to Treatment and Competence Scale、 Joint Engagement	Researchers
Shire et al. 2016	USA	43	42	Individual courses	10w	10 h	Individual courses	Joint Engagement	Researchers
Kasari et al. 2015 [12]	USA	43	43	Individual courses	10w	10 h	Individual courses	RDLS	NP
Shire et al. 2022	USA	26	30	Individual courses + Group	12w	24 h	Individual courses + Group	ESCS	Researchers
Sterrett et al. 2022	USA	36	32	Individual courses + Group	8w	48 h	Group	MSEL、ADOS	NP
ESI vs. TAU									
Wetherby et al. 2006 [23]	USA	17	18	Individual courses	48w	NP	NP	CSBS	Researchers
ESI vs. PEI									
Wetherby et al. 2014	USA	42	40	Individual courses	36w	120 h	Group	MSEL、ADOS	Researchers
CPMT vs. TAU									
Alfieri et al. 2021	ITA	5	7	Individual courses	24w	27 h	NP	ESCS、VABS	Researchers
Valeri et al. 2020 [7]	ITA	17	17	Individual courses	24w	111 h	Individual courses	ADOS、MCDI	Researchers、Parents
Other NDBI									
Brian et al. 2016 [25]	CAN	30	32	Individual courses	12w	18 h	NP	MSEL、ADOS、PLS	Researchers
Wainer et al. 2021	USA	7	8	Individual courses	15w	NP	NP	RIT fidelity、SCC	Researchers、Parents
Manohar et al. 2019	IND	26	24	Individual courses	12w	NP	NP	CARS	Researchers

*NP/N *Not reported, *ESI *Early Social Interaction Project, *RIT *Reciprocal Imitation Training, *ESDM *Early Start Denver Model, *PRT *Pivotal Response Training, *ImPACT *Improving Parents as Communication Teachers, *Social ABC *Social Antecedent-Behavior-Consequences, *JASPER *Joint Attention, Symbolic Play, Engagement, and Regulation, *CPMT *Cooperative Parent-Mediated Therapy, *MSEL *Mullen Scales of Early Learning, *SRS *Social Responsiveness Scale, *SCQ *Social Communication Questionnaire, *VB-MAPP *Verbal Behavior Milestones Assessment and Placement Program,  *ADOS *Autism Diagnostic Observation Schedule, *PLS *Preschool Language Scales, *BIRS *Behavioral Intervention Rating Scale, *VABS *Vineland Adaptive Behavior Scales, *GDC-C *Griffiths Developmental Scales – Chinese, *CARS *Childhood Autism Rating Scale, *FEAS *Functional-Emotional Assessment Scale, *ESCS *Early Social Communication Scales, *CSBS *Communication and Symbolic Behavior Scales, *MCDI *MacArthur-Bates Communicative Development,  *SCC *Social communication checklist, *PIT Fidelity *Project ImPACT for Toddlers–Parent Intervention Fidelity, *RDLS *Reynell Developmental Language scales

### Characteristics of the included studies

These studies were published between 2006 and 2023. They had a combined sample size of 1743 participants, ranging in age from 6 months to 60 months old with a median age of 39.06 months (SD = 14.08). Of the 32 studies included, 21 were conducted in the USA, 6 in China, 1 in Ireland, 1 in Canada, 1 in India, and 2 in Italy. The major characteristics of the included studies is presented in Table 2.

**Fig. 2:**
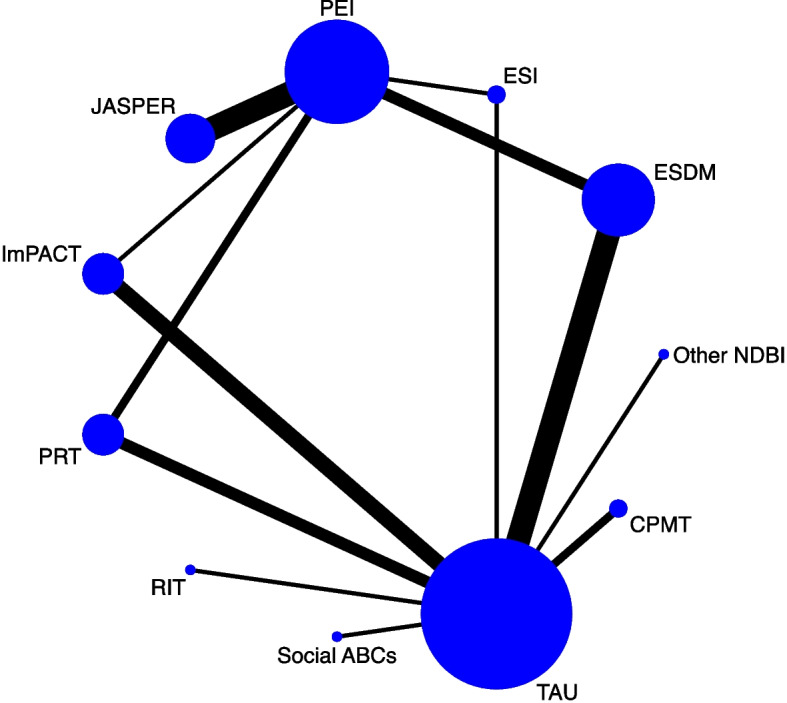
The overall network map of different NDBIs

The NDBI subgroups included in this study were PRT (*n* = 5), ImPACT (*n* = 5), RIT (*n* = 1), JASPER (*n* = 6), ESDM (*n* = 9), ESI (*n* = 2), Social ABCs (*n* = 1) and CPMT (*n* = 2) and other NDBI(*n* = 1). Different parent training methods included individual courses (*n* = 23), group(*n* = 4), Individual courses plus group(*n* = 5). The mean (SD) duration of intervention was 21.34 ± 23.00 weeks. Children’s outcomes were mainly assessed using developmental scales (Mullen, GDS, VB-MAPP、VABS); and scales assessing autistic traits (ADOS-II, CARS, SRS, SCQ). The parents’ fidelity scales were based on the EMDS, ImPACT, RIT, or self-made scales. The majority of the selected assessment tools were administered by professional evaluators or individuals actively participating in the research, who are collectively referred to as “researchers” in this study. Assessment tools that include parental reports were used in only five studies. The overall network map of different NDBIs is shown in Fig. 2.

### Risk of bias in the included studies

**Fig. 3:**
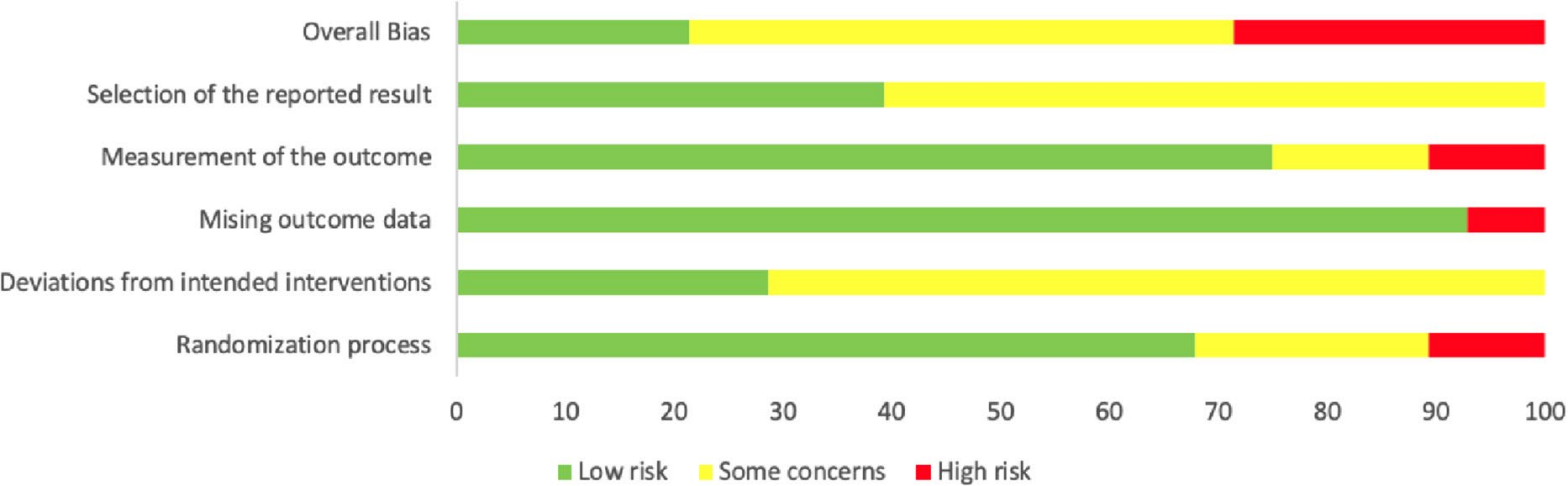
The overall risk of bias of included studies

During the randomization process, three studies were deemed high-risk due to the absence of randomization [32, 37, 36]. In terms of outcome measurement, three studies were considered high-risk: one due to parental completion of assessments [35], and the other for the use of self-made scale [34] and reliance on a single scale throughout the research [23]. The remaining studies were categorized as low risk or presented some concerns. The overall risk of bias assessment for the included studies is depicted in Fig. 3. Specific results of risk of bias and publication bias can be seen in Appendix S2 and S3.

### Parent fidelity

This meta-analysis revealed that most interventions exhibited a significant difference in parent fidelity between trained and untrained parents (SMD = 1.67, 95% CI: 0.74 to 2.61). However, the intensity of training, whether high or low, did not yield significant differences in parent fidelity (SMD = 0.97, 95% CI: -0.01 to 1.95). For instance, the ImPACT intervention significantly improved parent fidelity compared to TAU (SMD = 0.90, 95% CI: 0.39 to 1.42) and PEI (SMD = 1.10, 95% CI: 0.28 to 1.91). Similarly, the RIT intervention showed positive outcomes in parent fidelity (SMD = -3.32, 95% CI: 1.60 to 5.03). Additionally, the Social ABCs group exhibited significantly higher parent fidelity than the TAU group (SMD = 4.02, 95% CI: 3.14 to 4.91). Conversely, no significant difference in parental fidelity was observed with the ESDM interventions (SMD = 0.91, 95% CI: -0.03 to 1.85); however, the level of fidelity varied significantly with the training’s intensity high or low (SMD = 1.42, 95% CI: 0.76 to 2.09). In the PRT intervention, a notable difference in parent fidelity was observed between lower and higher intensity PEI (SMD = 3.53, 95% CI: 2.26 to 4.79). However, In the case of the JASPER intervention, increasing training intensity did not improve parent fidelity (SMD = -0.26, 95% CI: -0.76 to 0.25). A detailed description of parent fidelity is provided in Table 3.

### Language skills of children with ASD

Overall, there is a difference in the development of children’s language skills between parents who receive training and those who do not (SMD = 0.40, 95% CI: 0.15, 0.65). However, the intensity of the training—whether more or less intensive—does not affect the development of these skills (SMD =-0.02, 95% CI: -0.33, 0.29). Notably, a significant difference is observed only when the ESDM is employed, distinguishing between trained and untrained parents (SMD = 0.41, 95% CI: 0.04 to 0.79).

### Social skills of children with ASD

Regarding children’s social skills, we found a statically significance difference on whether parents have received training (SMD = 0.49, 95% CI: 0.18, 0.80) and the level of training intensity, whether high or low (SMD = 0.41, 95% CI: 0.07, 0.74). However, in the context of common NDBI, no statistically significant differences were observed. Conversely, within the realm of uncommon NDBIs, ESI (SMD = 0.70, 95% CI: 0.01, 1.38) and RIT (SMD = 0.49, 95% CI: 0.18, 0.80) have demonstrated notable efficacy.

### Motor skills of children with ASD

In terms of motor skills development, there is a notable overall difference between children of parents who received training and those who did not (SMD = 0.48, 95% CI: 0.21, 0.74). In this study, a significant difference in children’s motor skills was observed in the context of the ESDM training (SMD = 0.44, 95% CI: 0.09 to 0.79). However, when PRT (SMD = 0.46, 95% CI: -0.09 to 1.01) or ESI (SMD = 0.63, 95% CI: -0.05 to 1.31) were implemented, the differences in motor skills development were not statistically significant.

### Ranking the parent-mediated NDBIs in different developmental domains

PRT emerged as the top-ranked intervention across several domains: it achieved the highest scores in social skills (SUCRA, 74.1%), language skills (SUCRA, 88.3%), and parent fidelity (SUCRA, 99.5%). ESDM ranked second in these domains, with scores of (SUCRA, 67.3%) in social skills, (SUCRA, 67.5%) in language skills, and (SUCRA, 63.8%) in parent fidelity. ImPACT and JASPER were closely matched as the third highest-ranking interventions, with scores in social skills (SUCRA, 60.7%; 54.0%, ), language skills (SUCRA, 35.4% and 36.5%), and parent fidelity (SUCRA, 43.5% and 48.4%).

Subsequent to a sensitivity analysis, the overall forest maps remained relatively stable, though there were some shifts in the rankings across domains. Due to the limited number of studies in some Naturalistic Developmental Behavioral Interventions (NDBI) subgroups, and because not all outcomes covered every domain, we excluded the SUCRA values of less common NDBIs from the main domains in our post-analysis refinement. This step was taken to minimize potential errors in the study. Detailed forest maps, SUCRA maps, and the results of the sensitivity analysis are presented in Appendix S4 and Appendix S5. For a comprehensive breakdown of these findings, please refer to Table 4.

**Table 3:** Combined effect values of different NDBI in different domains of children and parents

	Group		Language skills *SMD (95% CI)*	Social skills *SMD (95% CI)*	Motor skills *SMD (95% CI)*	Parent fidelity *SMD (95% CI)*
**NDBI**		baseline	-0.07 (-0.19, 0.05)	-0.09 (-0.20, 0.02)	-0.22 (-0.47, 0.03)	-0.05 (-0.3, 0.2)
	TAU vs NDBI	endpoint	0.40* **(0.15, 0.65)**	0.49* **(0.18, 0.80)**	0.48* **(0.21, 0.74)**	1.67* **(0.74, 2.61)**
	PEI vs NDBI		-0.02 (-0.33, 0.29)	0.41* **(0.07, 0.74)**	-	0.97 (-0.01, 1.95)
**NDBI subgroups**						
**Common NDBI**		endpoint				
PRT	TAU vs PRT		0.83 (-0.06, 1.72)	0.57 (-0.05, 1.19)	0.46 (-0.09, 1.01)	-
	PEI vs PRT		0.17 (-0.40, 0.75)	0.48 (-0.10, 1.06)	-	3.53* **(2.26, 4.79)**
ESDM	TAU vs ESDM		0.41* **(0.04, 0.79)**	0.46 (-0.14, 1.07)	0.44* **(0.09, 0.79)**	0.91 (-0.03, 1.85)
	PEI vs ESDM		-	-	-	1.42* **(0.76, 2.09)**
ImPACT	TAU vs ImPACT		0.08 (-0.21, 0.36)	0.32 (-0.10, 0.73)	-	0.90* **（** **0.39, 1.42** **）**
	PEI vs ImPACT		0.15 (-0.61, 0.91)	0.66 (-0.12, 1.44)	-	1.10* **(0.28, 1.91)**
JASPER	TAU vs JASPER		-	-	-	-
	PEI vs JASPER		-0.18 (-0.60, 0.25)	0.36 (-0.08, 0.81)	-	-0.26 (-0.76, 0.25)
**Uncommon NDBI**		endpoint				
ESI	TAU vs ESI		-	0.70* **(0,01, 1.38)**	0.63 (-0.05, 1.31)	-
CPMT	TAU vs CPMT		0.67 (-0.31,1.65)	0.48(-0.69, 1.65)	-	-
RIT	TAU vs RIT		-	0.49* **(0.18, 0.80)**	-	3.32* **(1.60, 5.03)**
Social ABCs	TAU vs Social ABCs		-	-	-	4.02* **(3.14, 4.91)**

Significant results are in bold and *

**Table 4:** SUCRA values of NDBIs in main domains

Domains	Treatment	SUCRA	Domains	Treatment	SUCRA
Social skills	PRT	74.1	Parent fidelity	PRT	99.5
	ESDM	67.3		ESDM	63.8
	ImPACT	60.7		JASPER	48.4
	JASPER	54.0		ImPACT	43.5
Language skills	PRT	88.3			
	ESDM	67.5			
	JASPER	36.5			
	ImPACT	35.4			
Motor skills	PRT	74.5			
	ESDM	72.8			

*SUCRA *The surface under the cumulative ranking curve

## Discussion

The purpose of the current study was to evaluate the efficacy of various NDBIs across multiple domains: children’s language, social and motor skills, and parental fidelity. Initially, the effectiveness of different NDBIs was compared against the TAU group to ascertain their relative impact across these domains. Subsequently, a comparison with the PEI group was conducted to assess variations in intervention intensity for parents. Finally, the study aimed to rank the different intervention methods based on their effectiveness in each respective domain.

The analysis revealed that ImPACT is more readily operationalized by parents in terms of achieving fidelity. In contrast, PRT and the ESDM necessitate a heightened intensity of parent training to attain comparable levels of fidelity. Specifically, parent-mediated ESDM demonstrates notable improvements in language and motor skills among children with ASD. Furthermore, when administered with sufficient training intensity, parent-mediated PRT shows promising potential in enhancing children’s language abilities, social interactions, and motor skills.

Recent research suggests that high-fidelity parent implementation of intervention combined with frequent opportunities for results in the greatest child gains [26, 38]. A study of parent fidelity in P-ESDM showed that only about half of the studies met the criteria for fidelity [39], in this study, there was no significant difference between parents who received ESDM training and the TAU group. This may be because the ESDM system emphasizes that parents only assist in enhancing the effects of the therapist’s intervention [13], so the intensity of parent training may not be enough, however, significant differences can be seen in ESDM training for parents under large or small intervention intensity, which once again proves that the original parent training intensity of ESDM is not enough. In ImPACT, good parent fidelity is shown, which may be related to its flexible online course model and complete teaching manual [21]. After indirect comparison, this study concluded that PRT is a more effective method to improve parent fidelity than ImPACT. The pace and difficulty level of teaching of PRT are constantly individualized based on a child’s skills and motivation, and the instructional cues and materials are varied to help children broaden their attention and generalize learning from the outset [40], so PRT is difficult to understand immediately. Our research shows that parents who receive higher-intensity PRT training show better fidelity, which is contrary to the study of Svetlana [41]. In their study, PRT was used to train parents in specific language skills, which cannot fully convince researchers that PRT’s short-term parent training can achieve good fidelity among parents in all domains of children. We believe that more intensive PRT training is needed for parents to achieve fidelity standards. According to current research, JASPER is not the best choice for improving parent fidelity. RIT and Social ABCs have shown the potential to improve parent fidelity. This may be because RIT only emphasizes imitation [24], which is easy for parents to understand, while Social ABCs emphasizes step-by-step real-time teaching [25], making it easier for parents to combine theory and practice.

With growing globalization, interconnectedness, and complexity of our societies, social skills have become increasingly important which not only promotes good cooperation, but also helps us achieve good mental health [42]. However, social impairment is the core defect of ASD, and it is difficult to fundamentally improve it [1]. In this study, overall parent-mediated NDBI can enhance the social skills of children with ASD, which is consistent with the meta-analysis results of Micheal Sandbank [27]. In the meta-analysis of each NDBI methods, significant effects cannot be directly seen, which may still be related to the risk of bias in studies. Through indirect comparison, the best way to improve the social skills of children with ASD through parent training is PRT.

The World Health Organization has identified language as 1 of the domains of development that is associated with not only early learning and academic success but also economic participation and health across the lifespan [29]. Among children with ASD, many, except Asperger children, have language delays [43]. In terms of children’s language skills, this study shows that parent-mediated ESDM has a good effect. This result is consistent with Elizabeth’s review study [15]. A meta-analysis showed that PRT can significantly improve the language skill of children with ASD [17], in our research, when compared with various NDBIs, parent-mediated PRT was the best method to improve language function in children with ASD, while in direct comparison of control group, it cannot directly reflect its superiority in improving language in children with ASD, which may have something to do with parents’ accumulation of professional knowledge, and further research is needed.

Motor coordination deficits are commonly found in people with ASD [44]. The most critical one is the integration disorder of motor and social information [45]. ESDM has detailed gross and fine motor development milestone targets, and emphasizes the coordination of eyes and movements [46]. In our research, ESDM showed good efficacy in improving the motor skills of children with autism. In indirect comparison, PRT showed better efficacy than ESDM, and further direct demonstration is needed in follow-up studies.

In general, the quality of most of the included studies was relatively high, while heterogeneity was low. Readers should, however, be aware of the following limitations when interpreting the results of this study: There are few studies on uncommon NDBIs, and a large number of studies are needed to demonstrate their effects in various domains; Another issue that requires attention is the diversity of the measures used to evaluate intervention outcomes. For autistic children, proximity and boundedness of outcome cannot be ignored. Outcomes that were coded as proximal to the intervention tends to have significantly larger effects than those that were coded as distal. Compared to context-bound outcomes, the effect sizes were usually smaller for outcomes coded as generalized or potentially context-bound [5]. Moreover, the evaluation results reported by some parents may lack objectivity due to parents’ insufficient understanding for children’s normal development and behavior [4]. For parents, a unified standard is needed to put into practice for the evaluation of the parent fidelity of NDBI, so as to compare the efficacy between different parent-mediated NDBI [6].

## Conclusions

In conclusion, this study demonstrated that parent-mediated ImPACT interventions are effective in achieving high fidelity among parents, positioning them as a suitable initial intervention for children recently diagnosed with ASD. In the subsequent phase of parent training, parent-mediated ESDM has been shown to enhance language and motor skills in children with ASD. Finally, with adequate training intensity, parent-mediated PRT shows potential for further enhancements in language, social, and motor skills. This positions it as an integral third stage in a structured and comprehensive parent training program for children with ASD.

### Supplementary Information

**Supplementary Material 1.**

## Data Availability

The data used to support the findings of this study are included within the article.

## Abbreviations

ASD: Autism spectrum disorder
NDBI: Naturalistic developmental behavioral intervention
RCTs: randomized controlled trials
SUCRA: Surface under the cumulative ranking curves
Project AIM: Project Autism Intervention Meta-analysis
NMA: Network meta-analysis
CNKI: China National Knowledge Infrastructure
TAU group: The treat as usual group
PEI group: The parent education intervention group
RoB: The risk of bias
PRT: Pivotal response treatment
ESDM: Early start denver model
ABA: Applied behavior analysis
ImPACT: Improving parents as communication teachers
JASPER: Joint attention, symbolic play, engagement, and regulation
ESI: Early social interaction
CPMT: Cooperative Parent-Mediated Therapy
RIT: Reciprocal Imitation Training
MSEL: Mullen Scales of Early Learning
SRS: Social Responsiveness Scale
SCQ: Social Communication Questionnaire
VB-MAPP: Verbal Behavior Milestones Assessment and Placement Program
ADOS: Autism Diagnostic Observation Schedule
PLS: Preschool Language Scales
BIRS: Behavioral Intervention Rating Scale
VABS: Vineland Adaptive Behavior Scales
GDC-C: Griffiths Developmental Scales – Chinese
CARS: Childhood Autism Rating Scale
FEAS: Functional-Emotional Assessment Scale
ESCS: Early Social Communication Scales
CSBS: Communication and Symbolic Behavior Scales
MCDI: MacArthur-Bates Communicative Development
SCC: Social communication checklist
PIT Fidelity: Project ImPACT for Toddlers–Parent Intervention Fidelity
