# Supplementary material for: Comparison of the efficacy of parent-mediated NDBIs on developmental skills in children with ASD and fidelity in parents: a systematic review and network meta-analysis
Source: BMC Pediatr. 2024 Apr 25;24:270. doi: 10.1186/s12887-024-04752-9 (PMC11044316; doi:10.1186/s12887-024-04752-9)

**Supporting information to the manuscript**

**Comparison of the efficacy of parent-mediated NDBIs on developmental skills in children with ASD and fidelity in parents: A systematic review and network meta-analysis**

S1. Search Strategy

S2. Risk of bias in the included studies

S3. Funnel plot of all studies

S4. Forest map

S5. SUCRA

S6. The inconsistency analysis (node-splitting method）

**S1. Search strategy**（Take PubMed search as an example）

((autism) or (autistic) or (Asperger) or (autism spectrum disorder) or (autistic disorder)) AND ((parent) or (parents) or (caregiver) or (caregivers) or (family)) AND ((randomized controlled trial) or (randomized clinical trial) or (randomized clinical trials) or (Clinical randomized controlled trial) or (Randomized controlled clinical trial) or (RCT)

**S2. Risk of bias in the included studies**


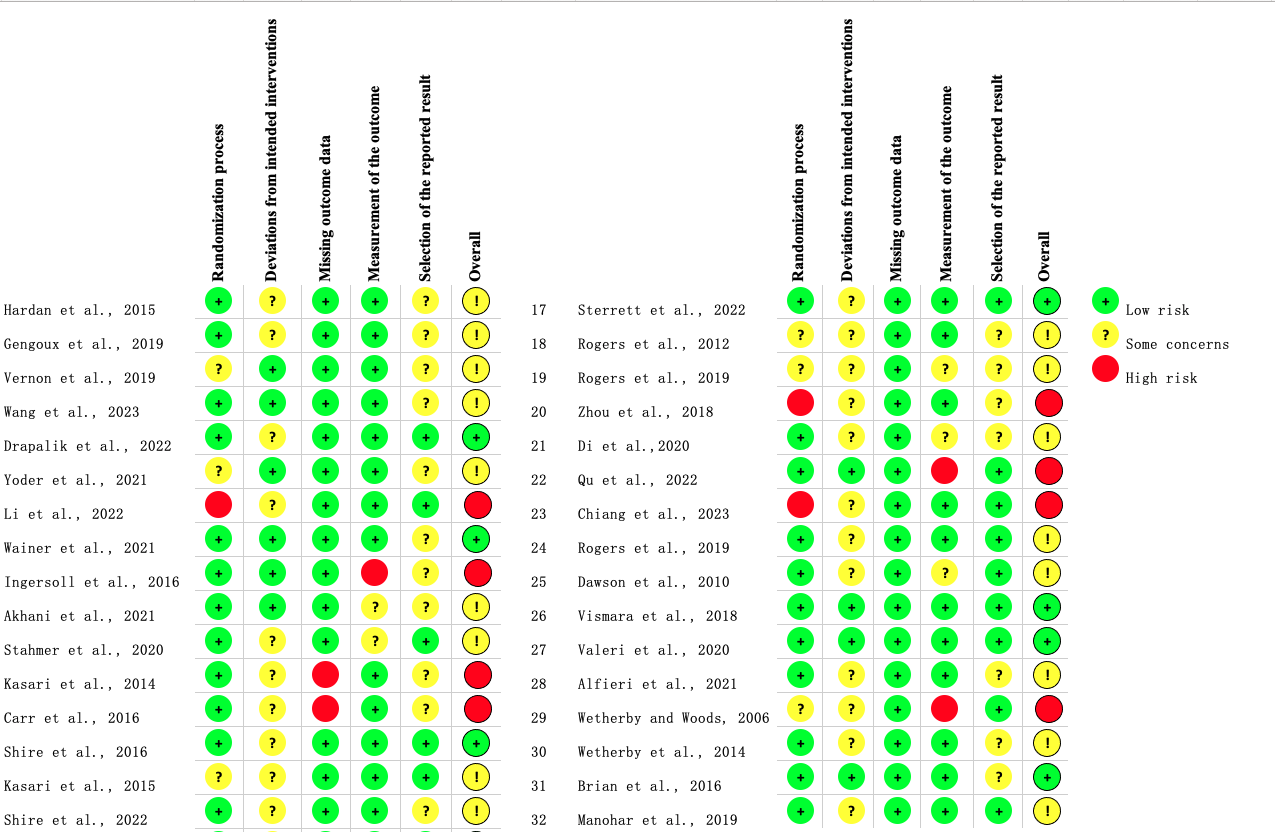


**S3. Funnel plot of all studies**

1. Social skills


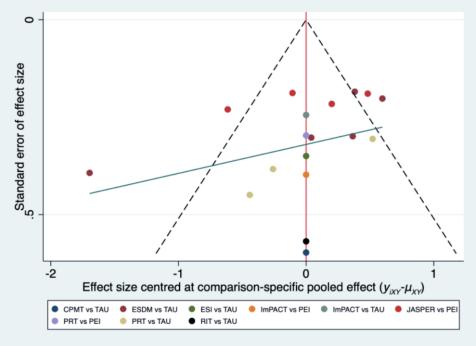


2. Language skills


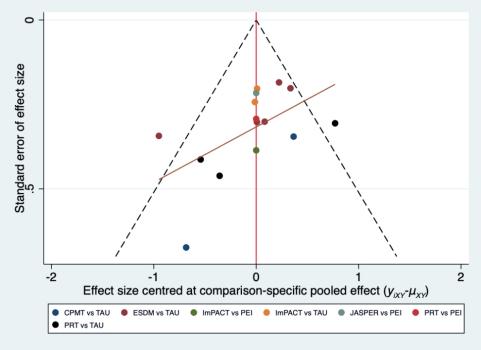


3. Motor skills


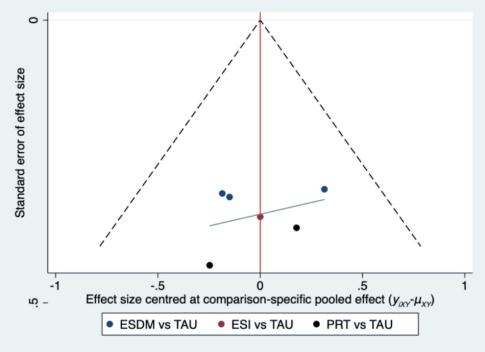


1. Parent fidelity


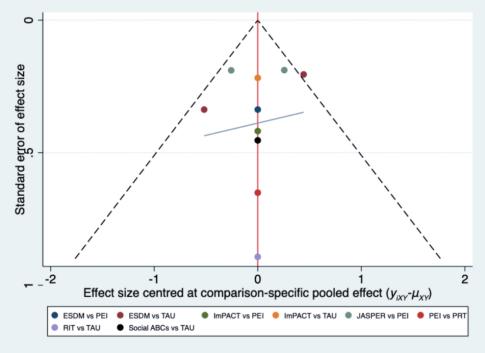


**S4. Forest map**

**1 Forest map at baseline in different domains**

1.1 Social skills

1.2 Language skills

1.3 Motor skills


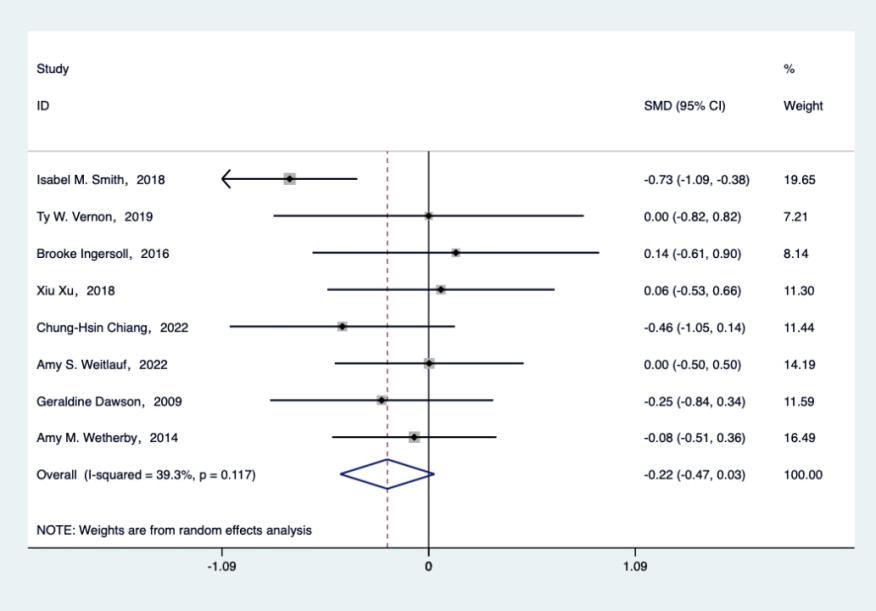


1.4 Parent fidelity


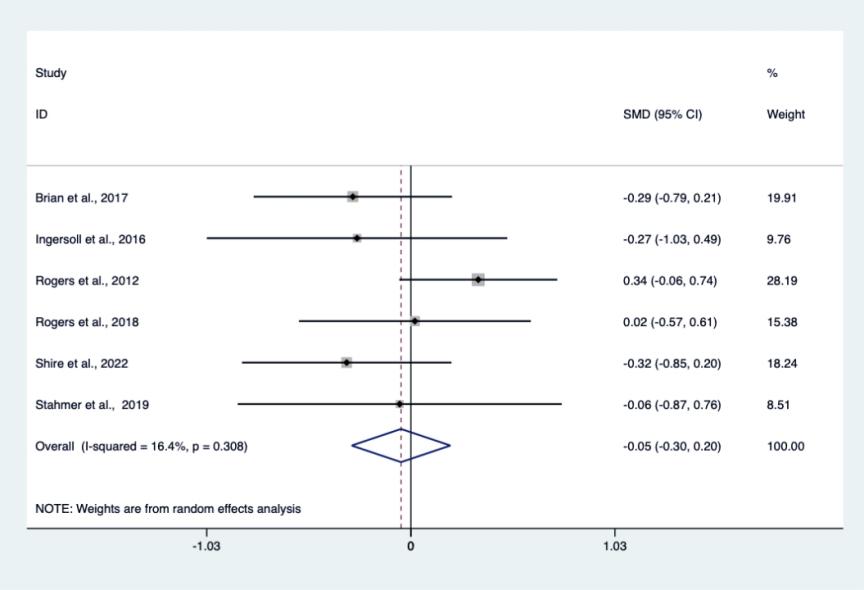


**2 Forest map at endpoint in different dimensions**

2.1 Social skills

TAU vs NDBI

Sensitivity analysis：

PEI vs NDBI


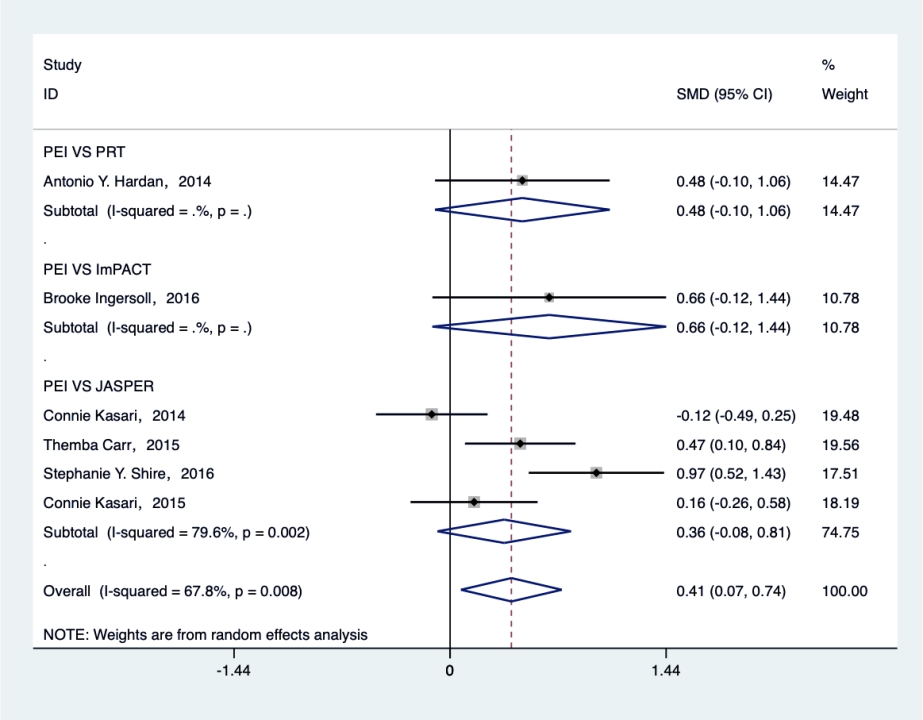


Sensitivity analysis：


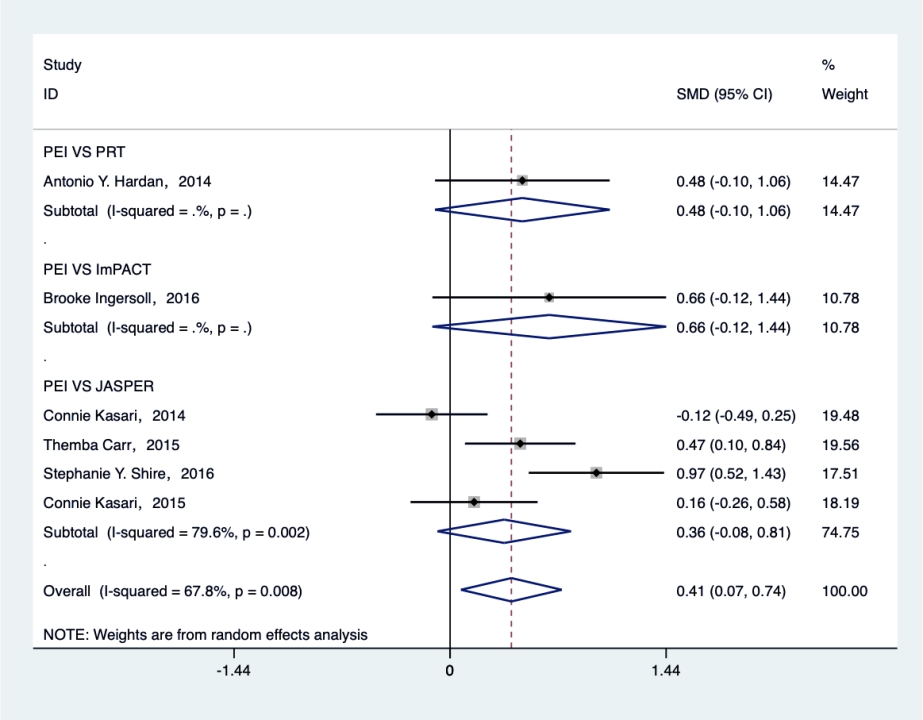


2.2 Language skills

TAU vs NDBI

Sensitivity analysis：

PEI vs NDBI


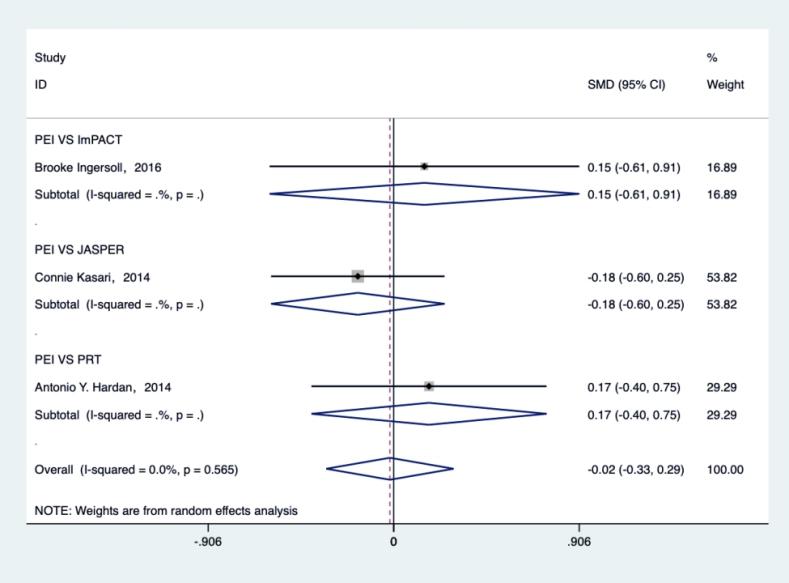


Sensitivity analysis：


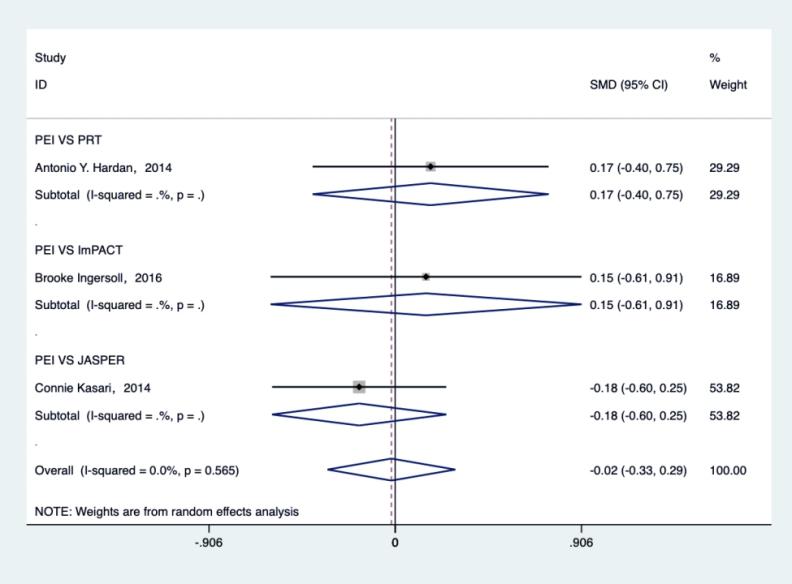


2.3 Motor skills

TAU vs NDBI


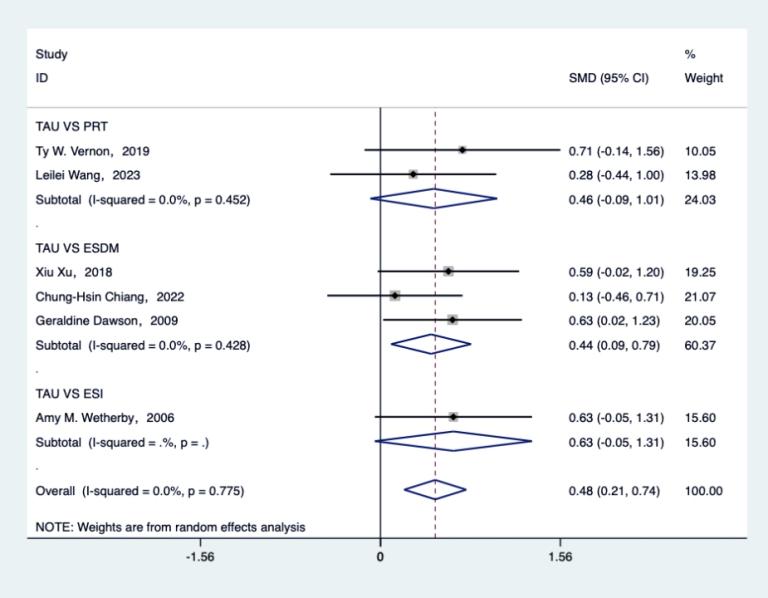


Sensitivity analysis:


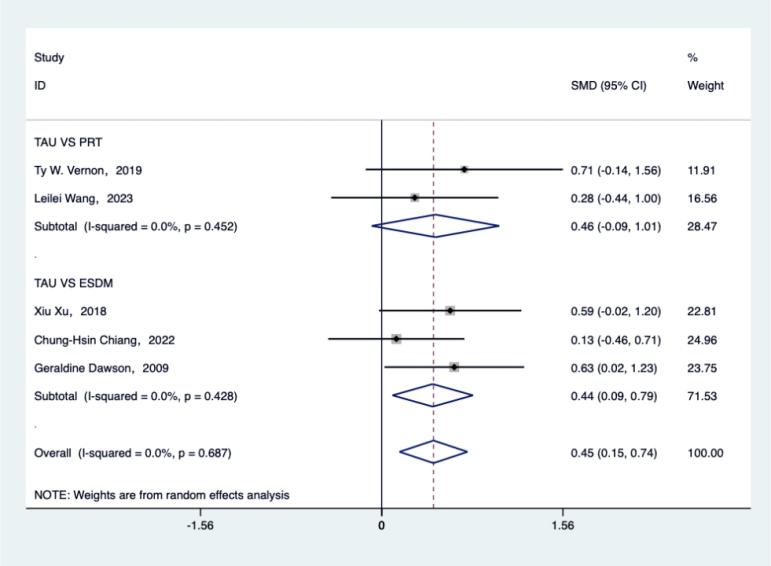


2.4 parent fidelity

TAU vs NDBI


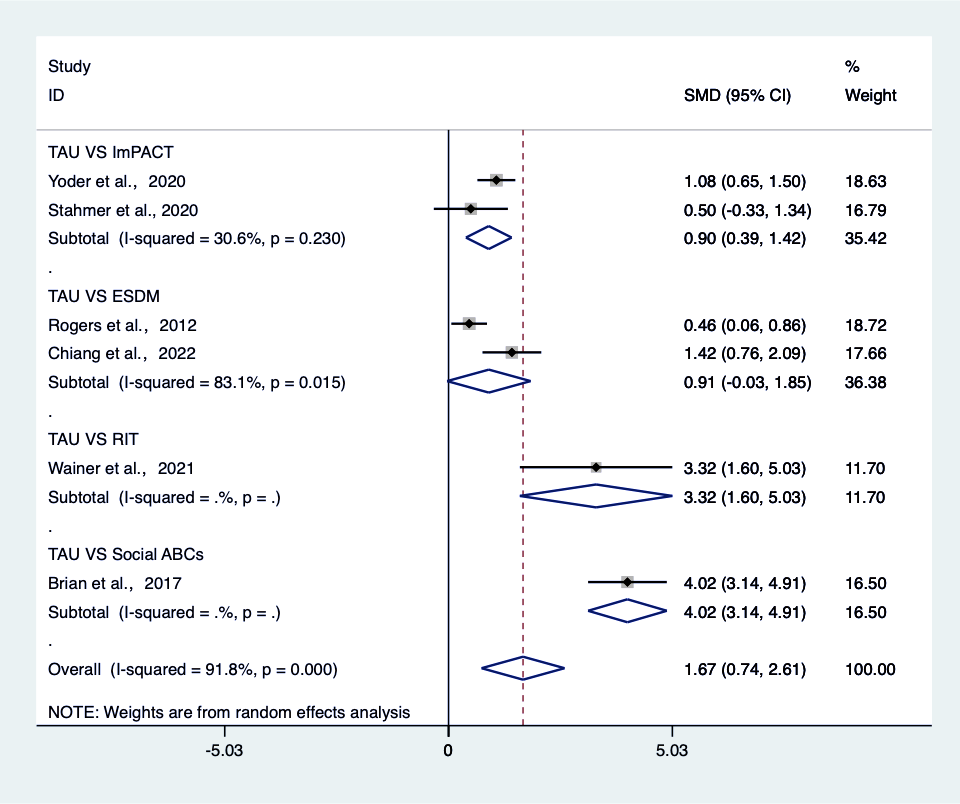


Sensitivity analysis：


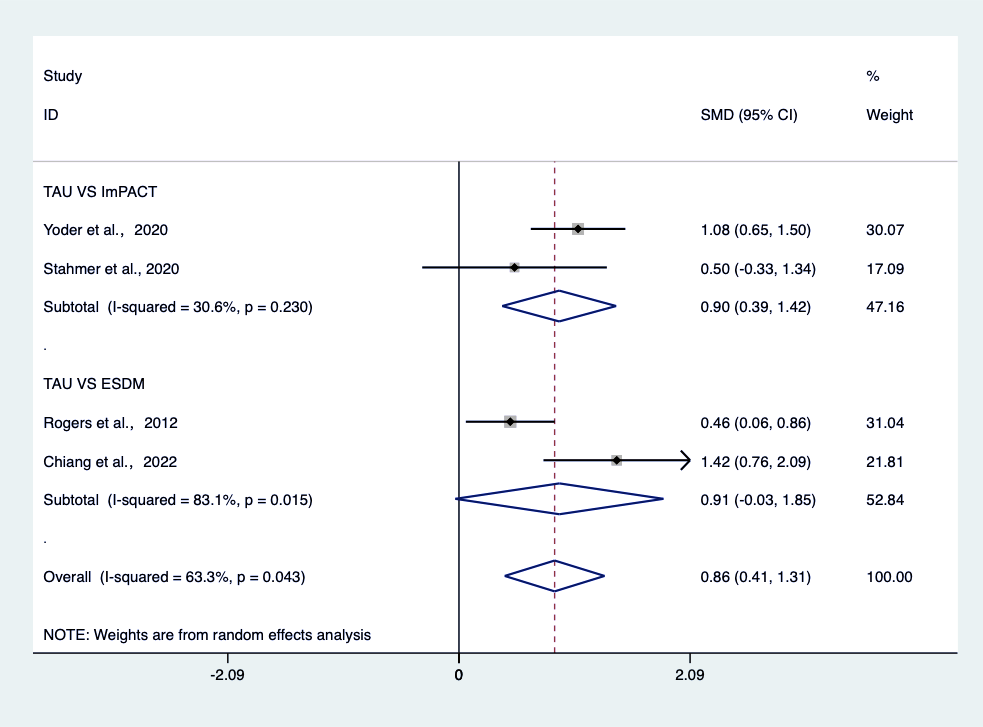


PEI vs NDBI


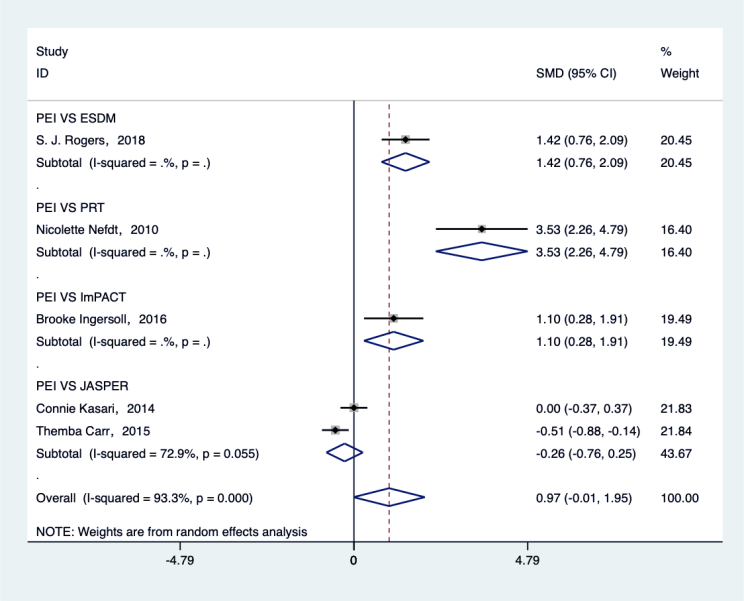


Sensitivity analysis：


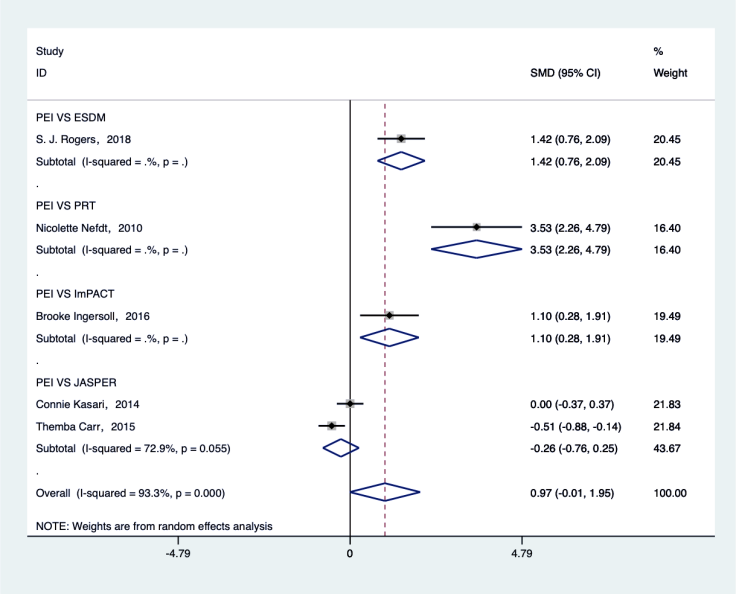


**S5. SUCRA**

1. **Language skills**

Sensitivity analysis:

**2. Social skills**

Sensitivity analysis:

**4. Motor skills**


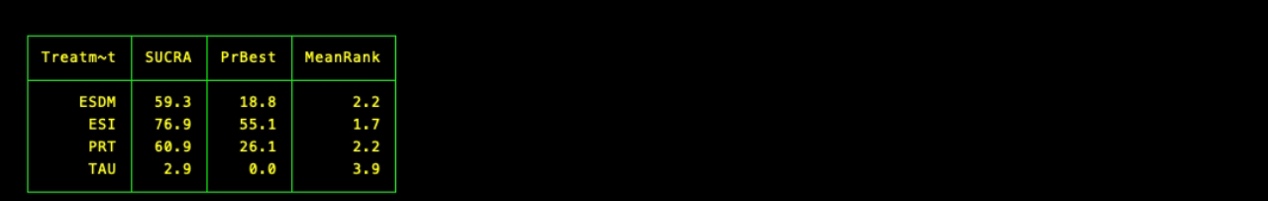


Sensitivity analysis:


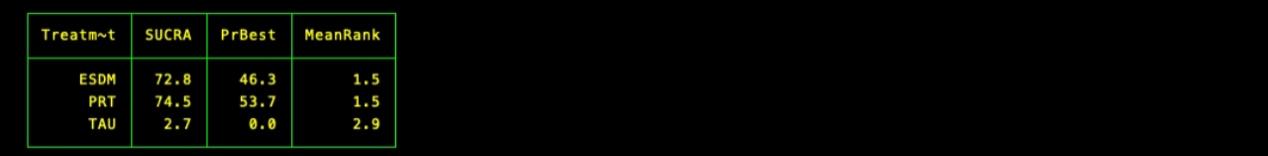


**4. Parent fidelity**


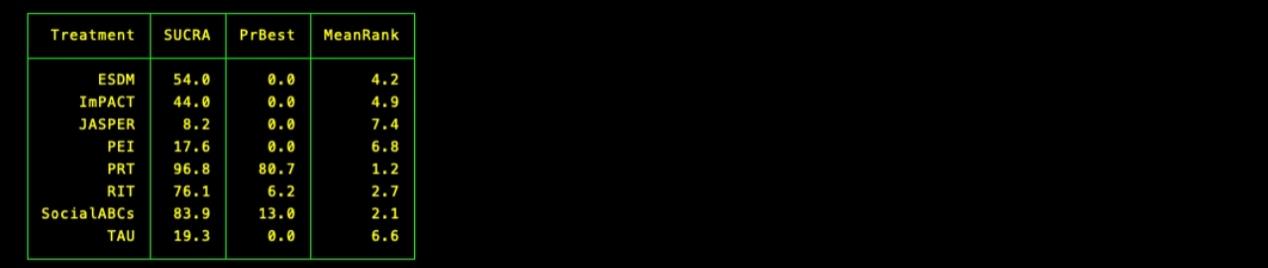


Sensitivity analysis：


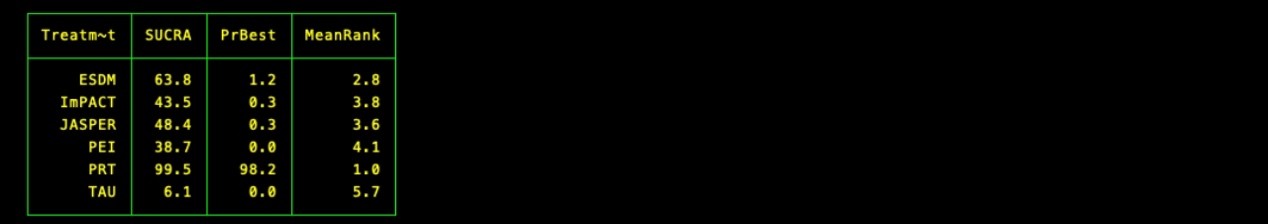


**S6. The inconsistency analysis (node-splitting method）**

1. Language skills


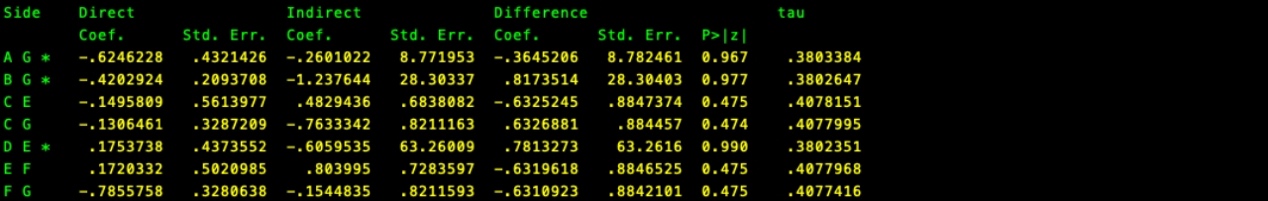


2. Social skills


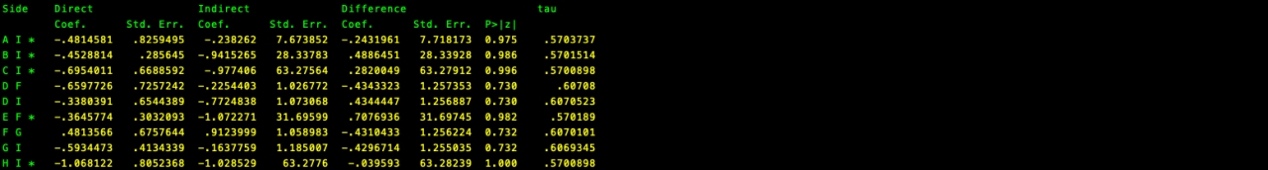


3. Motor skills


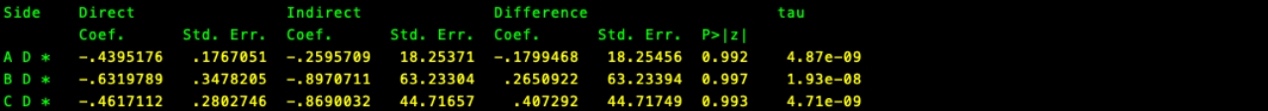


4. Parent fidelity


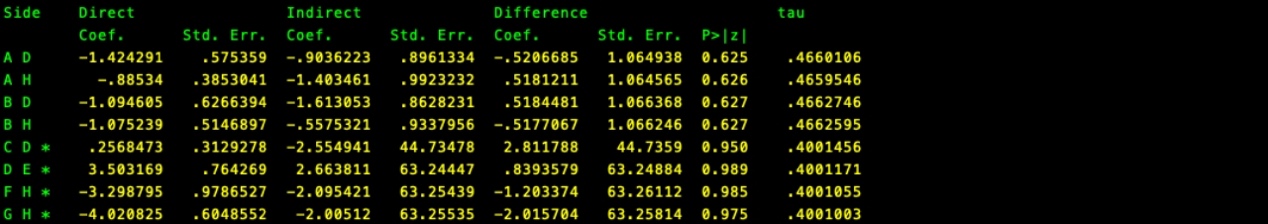

Supplement: Supplementary file 1 — Supplementary Material 1. [file 12887_2024_4752_MOESM1_ESM.docx]
